# Changes in health-related quality of life and inequality in China: evidence from a nationwide repeated cross-sectional survey (2021–2023)

**DOI:** 10.1007/s11136-025-04014-w

**Published:** 2025-07-16

**Authors:** Qiang Yao, Xiaodan Zhang, Siyuan Fan, Yibo Wu, Chaojie Liu

**Affiliations:** 1 School of Political Science and Public Administration, Wuhan University, Wuhan, Hubei China; 2 Centre for Social Security Studies, Wuhan University, Wuhan, Hubei China; 3 School of Public Health, Imperial College London, London, UK; 4 Department of Nursing, the Fourth Affiliated Hospital of School of Medicine, and International School of Medicine, International Institutes of Medicine, Zhejiang University, Yiwu, China; 5 School of Psychology and Public Health, La Trobe University, Melbourne, VIC Australia

**Keywords:** Health-related quality of life, HRQoL, China, Health inequality

## Abstract

**Background:**

Health improvement is a multidimensional process requiring attention to both health levels and equity. This study examined nationwide trends in both the levels and equality of health-related quality of life (HRQoL) of Chinese residents during the COVID-19 pandemic (2021–2023).

**Methods:**

Data were extracted from the Psychology and Behavior Investigation of Chinese Residents (PBICR) survey: 9,963 participants in 2021, 28,280 in 2022, and 45,003 in 2023. HRQoL was assessed using the EQ-5D-5 L. Changes in the weighted average EQ-5D-5L utility index (UI) and visual analogue scale (VAS) scores were assessed. OLS regression models were used to verify the changes in UI and VAS scores over time, controlling variations in other variables. The Erreygers Index (EI) was calculated to measure inequality in UI and VAS scores. RIF-EI-OLS models were employed to decompose contributors to EI scores.

**Results:**

There was a slight decline in HRQoL over the study period, with 2021 showing the highest weighted average UI score (0.9363 ± 0.1389), compared to 0.9332 (SD = 0.1524) in 2022 (β=-0.0081, *P* < 0.0001) and 0.9244 (SD = 0.1546) in 2023 (β=-0.0143, *P* < 0.0001). The EI value for UI in 2021 was 0.0703, showing a decreasing trend to 0.0635 in 2022 (β=-0.0105, *P* < 0.001), with no further significant change in 2023. Regional disparities in both UI and EI were evident. Higher socioeconomic status (e.g., income, education) was associated with higher UI and lower EI scores. Chronic conditions were associated with lower UI scores, while co-morbidity was associated with higher EI scores. Higher UI and lower EI scores were also associated with higher health literacy and a better family environment. The predictors of VAS were similar to those for UI. The EI value for VAS showed an increasing trend.

**Conclusions:**

From 2021 to 2023, residents in mainland China experienced a slight decline in HRQoL, accompanied by a lowered UI inequality but higher VAS inequality in 2022 and 2023. These may reflect how life and health change under the specific context of the COVID-19 pandemic.

**Supplementary Information:**

The online version contains supplementary material available at 10.1007/s11136-025-04014-w.

## Abstract (plain language summary)

The COVID-19 pandemic has had a profound impact on health, affecting both overall well-being and the equity of health distribution. This study explored changes in the health-related quality of life (HRQoL) among Chinese residents between 2021 and 2023, focusing on whether these changes were experienced equally across different population groups. The findings revealed a slight decline in HRQoL over this period, accompanied by lowered UI inequality but higher VAS inequality in 2022 and 2023. Wealthier individuals generally reported better health outcomes, underscoring a disparity linked to socioeconomic factors.

**Supplementary Information:**

The online version contains supplementary material available at 10.1007/s11136-025-04014-w.

## Introduction

Health is the indispensable prerequisite for the overall well-being of people and the foundation of economic and social development. In 2015, all 193 member states of the United Nations (UN) unanimously adopted the “2030 Agenda for Sustainable Development”, which aims to achieve 17 Sustainable Development Goals (SDGs) [1]. Health has a central place in SDG three: Ensure healthy lives and promote well-being for all at all ages [2]. Almost all of the other 16 goals are either directly related to health or contribute to health indirectly, encouraging countries to commit to improvements and advancements in the field of health. In 2016, China proposed the “Healthy China 2030” blueprint, with the core objective of raising health levels and regarding public health as a prerequisite for all future economic and social development.

However, the process of health improvement is not a single-dimensional linear advancement, but a multidimensional process that requires simultaneous attention to both health levels and health equity [3]. This means that while pursuing the overall improvement of health levels, it is also essential to ensure that everyone can equally access health services and resources. Universal health coverage emphasizes that everyone should be able to obtain the high-quality health services they need without financial hardship. This is not only about individual health rights but also the foundation for achieving overall improvements in societal health [2].

The World Health Organization (WHO) defines health as a state of complete physical, mental, and social well-being [4]. There are diverse health measurement indicators, including subjective and objective indicators. Objective indicators assess residents’ physiological and psychological parameters, including aspects such as physical function, treatment status, and medical history (e.g., mortality, morbidity). Subjective indicators reflect residents’ self-assessments of their health, typically represented in the form of self-rated health scores. With increasing emphasis on people-centered care, traditional health measurement indicators can no longer fully and accurately reflect health status [5]. The health-related quality of life (HRQoL) is a multifaceted concept that includes social functioning and physical and mental health status [6]. It is defined as ‘how well a person functions in their life and their perceived well-being in physical, mental, and social domains of health’ [7]. HRQoL, as a comprehensive method of evaluating health status, has played an irreplaceable role in recent years in clinical treatment, pharmaceutical research, preventive health care, and health economic evaluation. HRQoL has been widely used as a population health outcome measure in international literature [8, 9]. The EQ-5D family is perhaps the most commonly used instrument in assessing HRQoL [10].

Researchers have increasingly incorporated HRQoL into composite health metrics such as disability-adjusted life years (DALYs) to better capture population health outcomes [11]. The Global Burden of Disease (GBD) study has been instrumental in employing these indicators to explore causes of death, risk factors, and other determinants of health on a global scale [12]. However, significant gaps remain, particularly in integrating both health level and health equity perspectives. Most existing research focuses on single dimensions or equity alone, without a holistic approach. Research on health inequalities typically focuses on objective indicators such as disease prevalence [11, 13], BMI [14], and maternal and infant mortality rates [15], although there are emerging studies using subjective indicators like self-rated health [3].

This study addresses the gap in the literature by measuring changes in the level and equality of HRQoL in mainland China over the period of the COVID-19 pandemic from 2021 to 2023. The effect of the COVID-19 pandemic has been remarkable on a global scale. Global adult mortality rates markedly increased during the COVID-19 pandemic in 2020 and 2021, reversing previous decreasing trends [16]. However, there is a notable lack of research on recent HRQoL trends, especially the national trends associated with the COVID-19 pandemic [17, 18], despite some localized studies [19] in China.

## Methods

### Study setting

This study was conducted in mainland China during the COVID-19 pandemic. China adopted a phased approach to controlling the spread of the virus. From January to April 2020, strict containment measures were implemented, including a full lockdown of Wuhan. This was followed by a nationwide strategy of rapid responses to local outbreaks. In 2021 and 2022, China pursued a Dynamic Zero-COVID strategy, characterized by routine mass testing, the use of digital health codes, localized lockdowns, and strict quarantine protocols, all aimed at the early detection and containment of outbreaks. In December 2022, these measures were relaxed, and COVID-19 was downgraded to a Category B infectious disease, marking the end of the Dynamic Zero-COVID phase and the beginning of the country’s reopening. The year 2023 subsequently saw a surge in COVID-19 infections [20, 21].

### Design

This study follows the STROBE reporting guidelines for cross-sectional studies (Supplementary file S1). Data were extracted from the Psychology and Behavior Investigation of Chinese Residents (PBICR), a nationally representative annual survey conducted by the School of Public Health of Peking University, China [22, 23]. PBICR collected data regarding individual sociodemographic characteristics, personal health and behaviors, and family and social environments. Three waves of PBICR have been conducted: from July 10th to September 15th in 2021, from June 20th to August 31st in 2022, and from June 20th to August 31st in 2023.

### Data collection

PBICR data were collected through household visits conducted by data collection teams composed of higher-degree research students skilled in communication and teamwork. Each study participant was invited to self-complete the online survey through the platform Wen Juan Xing (https://www.wjx.cn/). Each team of investigators was responsible for collecting 100–200 questionnaires, with each team member contributing 30–90. Investigators were permitted to conduct household visits for data collection provided they held a green health code (indicating a negative nucleic acid test result) and the survey community was not under lockdown. For study participants who were under quarantine, data were collected through online video interviews. Eligible participants included those who were 12 years or older (restricted to 18 years or older in 2023), resided in the survey location permanently with no more than a one-month absence, and were able to understand the survey questions and provide informed consent. Those with serious mental health disorders, cognitive impairment, or prior involvement in other similar research projects were excluded.

### Population and sample

The PBICR adopted a multi-stage sampling strategy based on the principle of probability proportional to size (PPS), followed by random selection and quota allocation [22, 23]. Approximately 780 residential communities were selected from 202 districts or counties across 31 provinces and regions in mainland China (22 provinces, 4 centrally administered municipalities, and 5 autonomous regions).

In the first stage, capital cities and centrally administered municipalities were purposively included. Additionally, between 1 and 12 other cities within each province or autonomous region were randomly selected using a random number table, with the number of cities sampled proportional to the provincial population size. This PPS approach resulted in the selection of 120 cities in 2021, 148 cities in 2022, and 150 cities in 2023.

In the second stage, within each selected city, between 6 and 36 communities were randomly chosen, again using a random number table. The number of communities sampled in each city was determined by its population size, following the PPS principle, leading to the selection of approximately 780 to 800 communities overall.

In the third stage, a sampling quota was assigned to each community based on the overall sample size requirements and the resident population size of each community.

A total of 11,709 participants in 2021, 31,626 in 2022, and 48,485 in 2023 were recruited for the survey, with 40, 177, and 816 individuals refusing to provide informed consent, respectively. Logic checks excluded an additional 638 responses in 2021, 943 in 2022, and 1,839 in 2023 due to extremely short response times, logical inconsistencies, duplicate entries, and missing values in key data.

**Fig. 1:**
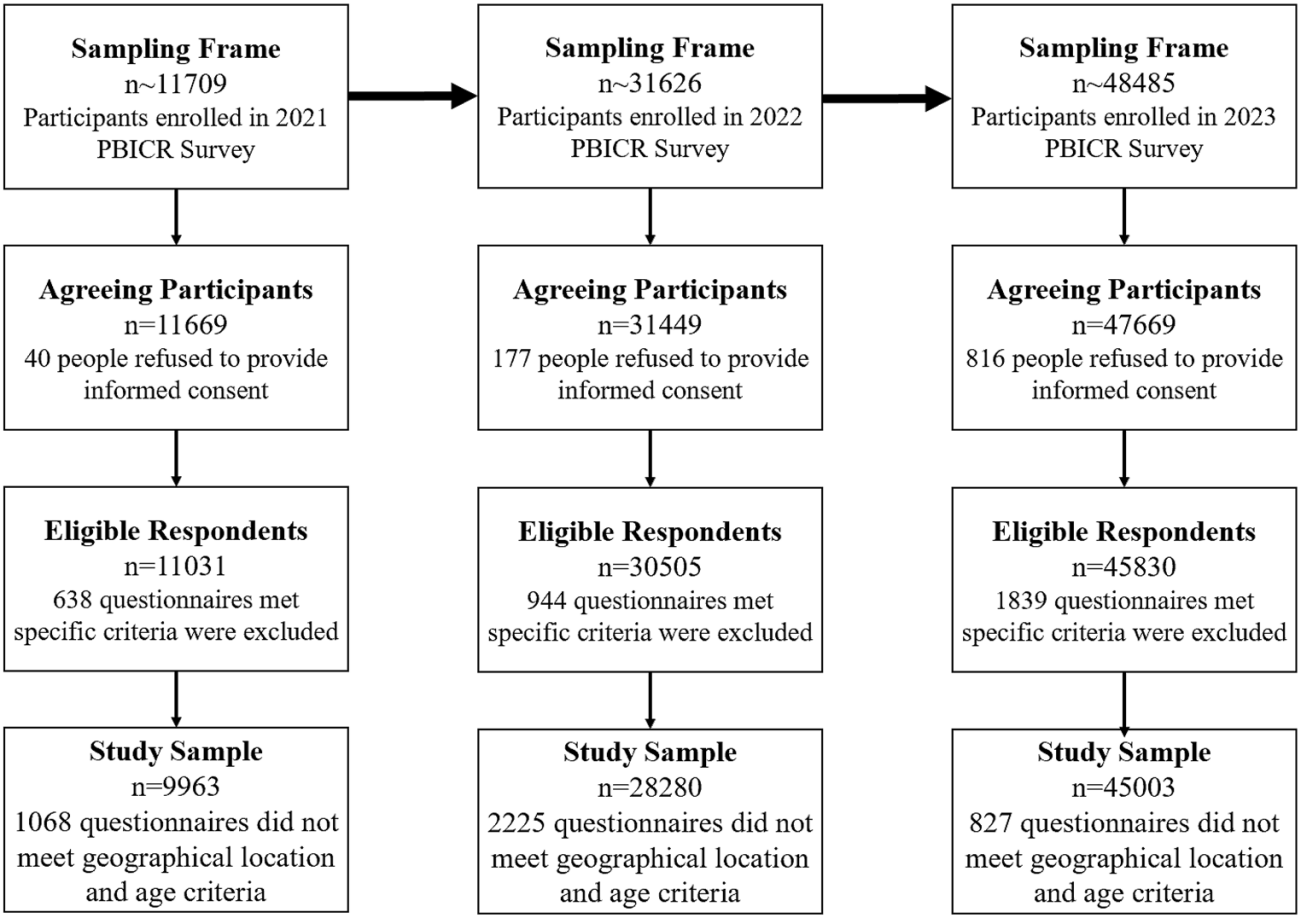
Participant recruitment and screening

To ensure comparability, the study restricted participants to those aged 19 years and older, as 18-year-old participants could not be identified separately in the 2021 dataset, and the 2023 survey was limited to individuals aged 18 years or older. This resulted in the removal of 1,065 responses in 2021, 2,161 in 2022, and 605 in 2023. Additionally, participants with incomplete address information or originating from outside mainland China were excluded (3 in 2021, 64 in 2022, and 222 in 2023). Consequently, the final sample included 9,963 participants in 2021, 28,280 participants in 2022, and 45,003 participants in 2023 for data analyses (Fig. 1). More details regarding the PBICR dataset are available in the metadata registry at https://wwwx-mol.com/groups/pbicr.

### Variables and measures

#### Dependent variable

HRQoL is the primary health outcome of interest in this study, which was measured by the EQ-5D-5 L developed by the EuroQol Group [24]. It is a brief, self-reported generic measure of current health that consists of five dimensions (Mobility, Self-Care, Usual Activities, Pain/Discomfort, and Anxiety/Depression), with each dimension rated on a five-level scale ranging from “no problem” to “extreme difficulty” [10]. Compared to its predecessor, the EQ-5D-3L, the EQ-5D-5L has stronger discriminatory power and better convergent validity with a lower ceiling effect [25, 26].

The reliability and validity of the Chinese version of EQ-5D instruments have been demonstrated, including a population value set for estimating the utility index (UI) [27, 28]. Efforts have been made to establish EQ-5D norms for general health status, and some studies have examined HRQoL inequalities in specific regions or populations [9, 29, 30].

The EQ-5D-5 L also contains a vertical EuroQol visual analog scale (EQ-VAS), ranging from 0 (the worst imaginable health) to 100 (the best imaginable health). And the EQ-VAS represents the respondents’ perspective to record their own self-rated health [10, 27].

#### Independent variables

The level and equality of HRQoL are determined by many factors, including biological, social, and economic factors [31, 32]. The action framework for social determinants of health identifies direct pathogenic factors, individual behaviors, and environmental influences as causes of health outcome disparities. This study categorized variables into seven distinct categories, based on the health determinants model [33, 34] and the health equity model [35], which addresses health disparities under different social contexts, such as political, economic, and cultural conditions.

1. **Individual demographic characteristics**: Gender (male, female) and age (19–30, 31–40, 41–50, 51–60, 61–70, 71 + years).
2. **Individual socio-economic characteristics**: Educational attainment (illiteracy or elementary, junior middle school, high school, tertiary education), marital status (unmarried, married, divorced/widowed), employment status (employed, student, retired, others), health insurance coverage (no, yes), and per capita household income (Chinese Yuan). Per capita monthly household income data were converted into quintile rankings within each province (lowest, lower, middle, higher, highest) to enable regionally adjusted data analysis.
3. **Individual health literacy**: Measured by the validated SF Health Literacy Survey questionnaire (low, medium, high) [22, 36].
4. **Individual health behavior**: Smoking (no, yes) and alcohol consumption (no, yes).
5. **Individual health status**: Chronic conditions measured by a checklist of diseases diagnosed by a doctor (0, 1, 2 or more), body mass index (BMI) (underweight: <18.5 kg/m^2^, normal: 18.5 kg/m²≤BMI < 24 kg/m², overweight: 24 kg/m²≤BMI < 28 kg/m², obesity: ≥28 kg/m²), and five personality traits (extraversion, agreeableness, conscientiousness, nervousness, openness) measured by the Big Five Inventory (10 items) [37].
6. **Family health**: Measured by the validated Family Health Scale-Short Form (very poor, poor, good, very good) [22, 38].
7. **Social support**: Measured by the validated Perceived Social Support Scale (low, medium, high) [22, 39] and residential environments indicated by region (eastern, central, western) and residency (rural, urban).

### Data analysis

To mitigate potential biases arising from the quota sampling method and nonresponses, the collected data were calibrated by adjusting for location, population size, sex, and age distribution. This approach not only ensures the generation of nationally representative data but also enhances comparability across different years. Previous studies have highlighted significant disparities in HRQoL between urban and rural populations in China [44, 45]. To account for these differences, age-gender-urban/rural weights, derived from the 2020 China Census data, were applied during data calibration (Supplementary File S2) for statistical analysis. All subsequent analyses, including the OLS regression models, the calculation of the EI, and the RIF decomposition, were conducted using these calibrated sampling weights to ensure unbiased and representative estimates.

### Level of HRQoL

Percentages of respondents reporting health problems were calculated. The Chinese value set developed by Luo et al. [28] was used to convert the reported problems into a utility index (UI) score for each respondent, which ranges from − 0.391 to 1.000, with 1.000 indicating the best possible health and a negative score indicating a state worse than death. The EQ-VAS was scored from 0 to 100, with a higher score indicating a higher level of perceived overall health. Weighted mean values and standard deviations of UI and EQ-VAS scores were calculated.

Ordinary Least Squares (OLS) regression models were developed to determine the factors associated with UI (or EQ-VAS) scores (y).

$$\eqalign{ y = {\beta _0} + {\beta _1}{X_1} + {\beta _2}{X_2} + {\beta _3}{X_3} + {\beta _4}{X_4} \cr + {\beta _5}{X_5} + {\beta _6}{X_6} + {\beta _7}{X_7} + {\beta _8}{X_8} + \varepsilon \cr} $$

where $\:{X}_{1}$ to $\:{X}_{7}$ indicate the seven categories of HRQoL determinants tested in this study, and $\:{X}_{8}$ represents the variable of year. $\:\epsilon\:$ stands for the error term.

To quantify the relative contributions of different determinants to the variation in HRQoL, we applied the Shapley decomposition method. Originally proposed by Lloyd Shapley, this approach decomposes the overall explanatory power of a regression model, as measured by the coefficient of determination (R²), into the individual contributions of each explanatory variable. It provides an equitable assessment of each variable’s importance in explaining the variance of the dependent variable by considering all possible permutations of variable entry into the model [40].

The Shapley decomposition method has been widely used in health inequality research [41]. In our analysis, it was applied to the OLS regression models of both EQ-UI and EQ-VAS scores to evaluate the contributions of various categories of HRQoL determinants, as well as the survey year, to the total explained variance.

### Inequality of HRQoL

Inequality of HRQoL was defined as systematic variations between socioeconomic groups in the EQ-UI and EQ-VAS indicators [42]. It was assessed using the Erreygers Index (EI), a widely recognized metric in health inequality research [43]. The EI, an adaptation of the traditional concentration index (CI) proposed by Erreygers [44], provides a more accurate reflection of inequality [44, 45]. It ranges from − 1 to + 1, where 0 indicates the absence of income-related inequality. A positive EI reflects pro-rich inequality, while a negative EI reflects pro-poor inequality [44, 46].

$$\:EI\left(h|y\right)=\frac{8}{{n}^{2}({Max}_{h}-{Min}_{h})}\sum\:_{i=1}^{n}{z}_{i}{h}_{i}$$

where $\:{h}_{i}$ and $\:{y}_{i}$ denote the UI (or EQ-VAS) score and income of the i-th individual, respectively. $\:n$ represents the sample size. $\:{Max}_{h}$ and $\:{Min}_{h}\:$represent the maximum and minimum scores of UI (or EQ-VAS) in the study population. $\:\sum\:_{i=1}^{n}{z}_{i}{h}_{i}$ expresses the rank-dependence character, which is a weighted UI (or EQ-VAS) sum of all individuals.

$\:{{Z}_{i}=\frac{n+1}{2}-{\lambda\:}_{i},\:\:where\:Z}_{i}$ and $\:{\lambda\:}_{i}$ represent the income rank deviation and the income rank of individual $\:i$, respectively. A positive sign of $\:{Z}_{i}$ indicates the group of the rich, while a negative sign of $\:{Z}_{i}$ indicates the group of the poor. A zero $\:{Z}_{i}$ means neither rich nor poor. The absolute value of the weight increase linearly with the distance of the rank from the middle [44].

This study employed the RIF-EI-OLS model to determine the marginal effect of each explanatory variable on HRQoL inequality. This model, a novel decomposition method proposed by Heckley in 2016, is derived from the influence function (IF) [47]. The RIF-EI-OLS method utilizes the recentered influence function (RIF) estimates of the health inequality index to determine the relationship between the RIF and explanatory variables. By establishing a regression function between the health inequality index and the explanatory variables, this method enables causal identification [43]. Compared to the traditional Wagstaff decomposition method, this method requires fewer and less restrictive assumptions. Meanwhile, this decomposition approach explains the causes of socioeconomic-related health inequality by directly decomposing the weighted covariance of health and socioeconomic rank, making estimation straightforward and results easy to interpret [47, 48].

The method involves two steps. The first step calculates the RIF (Recentered Influence Function) for the EI, which quantifies the contribution of each observation to the index. The RIF function uses the global statistic, EI, as a baseline and, through the influence function, facilitates a precise mapping from the aggregate measure to individual-level impacts. Specifically, the RIF for an individual observation with respect to the EI can be expressed as:

$$\:RIF\left({h}_{i};EI,F\right)=EI\left(h|y\right)+IF({h}_{i};EI,F)$$

$\:F$ is the distribution function of the health variable, and $\:IF({h}_{i};EI,F)$represents the influence function of the observation $\:{h}_{i}$ on the EI. By capturing the marginal contribution of individual observations to the EI, the RIF function bridges the global index and individual-level impacts, making it a powerful tool for inequality decomposition.

In the second step, the calculated RIF values are regressed on a set of explanatory variables using ordinary least squares (OLS) to estimate their marginal effects on the EI. The regression coefficients directly quantify how each explanatory variable contributes to health inequalities. The regression model is specified as:

$$\eqalign{\>RIF\left( {{h_i};EI} \right) = \gamma {\>_1}{X_1} + \gamma {\>_2}{X_2} + \gamma {\>_3}{X_3} + \gamma {\>_4}{X_4} \cr + \gamma {\>_5}{X_5} + \gamma {\>_6}{X_6} + \gamma {\>_7}{X_7} + \gamma {\>_8}{X_8} + \varepsilon {\>_i} \cr} $$

where $\:{X}_{1}$ to $\:{X}_{7}$ indicate the seven categories of EI determinants tested in this study, and $\:{X}_{8}$ represents the variable of year. γ is the vector of coefficients, representing the marginal effects of the covariates on the EI. ε stands for the error term.

### UI (or VAS) - EI composite index

The trade-off between equity and efficiency in health promotion has been a longstanding topic of academic inquiry. Two fundamental objectives of health systems are to maximize overall population health gains and to minimize unfair health inequalities [49]. In this study, we developed a composite index combining HRQoL (UI or VAS) and equity (EI), drawing inspiration from the universal health coverage framework [50]. To ensure consistency in interpretation, we transformed the equity component to 1−|EI|, so that higher values represent greater equity. This adjustment aligns the direction of change for both dimensions, allowing the composite index to range from 0 to 100, with higher scores indicating better population health and greater equity. This unified scale facilitates a clearer visualization of the balance between efficiency and equity across years. The formula is as follows:

$$\eqalign{\>UI\>\left( {or\>VAS} \right) - EI\>composite\>index \cr = \sqrt {\left( {1 - \left| {EI} \right|} \right)*100*UI\left( {or\>VAS} \right)*100} \cr} $$

### Data analysis

Frequency distributions and weighted means (standard deviations) were used to describe nominal/ordinal and continuous variables, respectively. All statistical analyses were conducted using Stata IC 16.0. The practical implementation of RIF-EI-OLS regression decomposition is straightforward with software such as Stata [48]. A p-value < 0.05 was considered statistically significant.

We present the findings related to the EQ-UI scores in the main document, while the EQ-VAS results are presented in the supplementary files.

### Ethical considerations

This study involved human participants and was approved by the Ethics Research Committee of the Health Culture Research Centre of Shaanxi (JKWH-2021-01/JKWH-2022-02) and Shandong Provincial Hospital (SWYX: NO.2023 − 198). Implied informed consent was obtained prior to each survey. Participants gave informed consent to participate in the study before proceeding to the online survey.

## Results

### Characteristics of respondents

The study sample was biased towards the most populous eastern developed zone (38.47%) and the least populated western underdeveloped zone (37.77%), compared to the central developing zone (23.76%). About 29.22% of respondents resided in rural areas (Table 1).

**Table 1:** Characteristics of study participants

Variables	2021		2021(weighted)		2022		2022(weighted)		2023		2023(weighted)		Total		Total(weighted)	
	*N*	%	*N*	%	*N*	%	*N*	%	*N*	%	*N*	%	*N*	%	*N*	%
Individual physiological characteristics																
Gender																
Male	4591	46.08	5035	50.54	12,162	43.01	14,305	50.58	20,197	44.88	22,764	50.58	36,950	44.39	42,025	50.48
Female	5372	53.92	4927	49.46	16,118	56.99	13,975	49.42	24,806	55.12	22,239	49.42	46,296	55.61	41,221	49.52
Age(years)																
19–30	3599	36.12	1880	18.87	13,012	46.01	5334	18.86	21,241	47.20	8489	18.86	37,852	45.47	15,765	18.94
31–40	1732	17.38	1947	19.55	3637	12.86	5530	19.55	6041	13.42	8800	19.55	11,410	13.71	16,245	19.51
41–50	2480	24.89	1957	19.64	4334	15.33	5555	19.64	7377	16.39	8840	19.64	14,191	17.05	16,367	19.66
51–60	1006	10.10	1899	19.07	2935	10.38	5393	19.07	5117	11.37	8582	19.07	9058	10.88	15,863	19.06
61–70	513	5.15	1326	13.31	2462	8.71	3762	13.30	2878	6.40	5987	13.30	5853	7.03	11,054	13.28
≥ 71	633	6.35	953	9.57	1900	6.72	2705	9.56	2349	5.22	4304	9.56	4882	5.86	7952	9.55
Individual socio-economic characteristics																
Educational attainment																
Illiteracy/elementary	1015	10.19	2105	21.12	3323	11.75	6250	22.10	4798	10.66	9697	21.55	9136	10.97	18,016	21.64
Junior middle school	1224	12.29	1885	18.92	3043	10.76	5070	17.93	5467	12.15	8976	19.95	9734	11.69	15,988	19.21
High school	1596	16.02	1712	17.18	6493	22.96	5837	20.64	9346	20.77	9194	20.43	17,435	20.94	16,781	20.16
Tertiary education	6128	61.51	4261	42.77	15,421	54.53	11,122	39.33	25,392	56.42	17,136	38.08	46,941	56.39	32,461	38.99
Marital status																
Unmarried	3297	33.09	2055	20.63	13,094	46.30	6693	23.67	21,668	48.15	11,071	24.60	38,059	45.72	19,851	23.85
Married	6224	62.47	7246	72.73	14,102	49.87	20,007	70.75	21,420	47.60	30,802	68.44	41,746	50.15	58,067	69.75
Divorced/widowed	442	4.44	662	6.65	1084	3.83	1580	5.59	1915	4.26	3131	6.96	3441	4.13	5327	6.40
Employment status																
Employed	4624	46.41	3713	37.26	9423	33.32	10,210	36.10	16,007	35.57	16,120	35.82	30,054	36.10	29,942	35.97
Student	2270	22.78	1331	13.36	10,196	36.05	4535	16.04	15,270	33.93	6582	14.63	27,736	33.32	12,512	15.03
Retired	883	8.86	1320	13.25	2886	10.21	3853	13.62	4487	9.97	7458	16.57	8256	9.92	12,613	15.15
Others	2186	21.94	3600	36.13	5775	20.42	9682	34.24	9239	20.53	14,843	32.98	17,200	20.66	28,179	33.85
Basic social health insurance																
No	2147	21.55	1997	20.04	2586	9.14	2216	7.84	3256	7.24	2766	6.15	7989	9.60	6993	8.40
Yes	7816	78.45	7966	79.96	25,694	90.86	26,064	92.16	41,747	92.76	42,237	93.85	75,257	90.40	76,253	91.60
Commercial medical insurance																
No	9746	97.82	9797	98.34	26,889	95.08	27,119	95.89	42,827	95.16	43,052	95.66	79,462	95.45	79,928	96.01
Yes	217	2.18	166	1.66	1391	4.92	1161	4.11	2176	4.84	1951	4.34	3784	4.55	3318	3.99
Household income ranking																
Lowest	3229	32.41	4094	41.09	7672	27.13	9463	33.46	12,319	27.37	14,406	32.01	23,220	27.89	27,806	33.40
Lower	1855	18.62	1804	18.11	5496	19.43	5939	21.00	9119	20.26	9649	21.44	16,470	19.78	17,499	21.02
Middle	1771	17.78	1630	16.36	5856	20.71	5287	18.69	8480	18.84	8336	18.52	16,107	19.35	15,266	18.34
Higher	1413	14.18	1192	11.96	4862	17.19	4283	15.15	7962	17.69	6987	15.53	14,237	17.10	12,455	14.96
Highest	1695	17.01	1243	12.48	4394	15.54	3308	11.70	7123	15.83	5625	12.50	13,212	15.87	10,220	12.28
Individual health literacy																
Health literacy																
Low	315	3.16	443	4.45	1467	5.19	1930	6.82	11,697	25.99	14,662	32.58	13,479	16.19	17,086	20.52
Medium	6308	63.31	6716	67.41	16,476	58.26	17,631	62.35	25,570	56.82	23,804	52.89	48,354	58.09	48,167	57.86
High	3340	33.52	2804	28.14	10,337	36.55	8719	30.83	7736	17.19	6537	14.52	21,413	25.72	17,993	21.61
Individual health behavior																
Smoking																
No	8583	86.15	8252	82.83	24,544	86.79	23,155	81.88	38,285	85.07	36,296	80.65	71,412	85.78	67,719	81.35
Yes	1380	13.85	1711	17.17	3736	13.21	5125	18.12	6718	14.93	8707	19.35	11,834	14.22	15,527	18.65
Alcohol drinking																
No	5768	57.89	5913	59.35	22,143	78.30	21,963	77.66	34,013	75.58	34,150	75.88	61,924	74.39	62,050	74.54
Yes	4195	42.11	4050	40.65	6137	21.70	6317	22.34	10,990	24.42	10,853	24.12	21,322	25.61	21,196	25.46
Individual health status																
Chronic conditions																
0	7959	79.89	7193	72.20	21,662	76.60	19,954	70.56	32,868	73.04	27,590	61.31	62,489	75.07	54,752	65.77
1	1347	13.52	1765	17.72	4465	15.79	5295	18.72	7426	16.50	9858	21.91	13,238	15.90	16,924	20.33
2ormore	657	6.59	1004	10.08	2153	7.61	3031	10.72	4709	10.46	7555	16.79	7519	9.03	11,570	13.90
Body mass index (BMI)																
Underweight	1232	12.37	1030	10.34	5099	18.03	3866	13.67	6220	13.82	4669	10.38	12,551	15.08	9578	11.51
Normal	6154	61.77	6045	60.68	16,808	59.43	16,748	59.22	27,772	61.71	27,542	61.20	50,734	60.94	50,393	60.54
Overweight	2175	21.83	2416	24.25	5119	18.10	6281	22.21	8842	19.65	10,530	23.40	16,136	19.38	19,174	23.03
Obesity	402	4.03	472	4.74	1254	4.43	1385	4.90	2169	4.82	2262	5.03	3825	4.59	4101	4.93
Extraversion	6.26 ± 1.58		6.23 ± 1.54		6.22 ± 1.63		6.18 ± 1.54		6.21 ± 1.60		6.20 ± 1.55		6.22 ± 1.61		6.20 ± 1.55	
Agreeableness	7.00 ± 1.49		6.97 ± 1.50		6.96 ± 1.48		6.95 ± 1.46		6.83 ± 1.41		6.86 ± 1.43		6.89 ± 1.45		6.90 ± 1.45	
Conscientiousness	6.91 ± 1.60		7.03 ± 1.58		6.67 ± 1.64		6.89 ± 1.60		6.64 ± 1.55		6.87 ± 1.54		6.68 ± 1.59		6.89 ± 1.57	
Nervousness	5.74 ± 1.49		5.69 ± 1.46		5.82 ± 1.56		5.71 ± 1.48		5.85 ± 1.44		5.75 ± 1.39		5.82 ± 1.49		5.73 ± 1.43	
Openness	6.41 ± 1.52		6.16 ± 1.49		6.53 ± 1.56		6.24 ± 1.49		6.35 ± 1.52		6.05 ± 1.47		6.42 ± 1.53		6.13 ± 1.48	
Family characteristics																
Family health																
Very poor	12	0.12	12	0.12	34	0.12	35	0.12	57	0.13	68	0.15	103	0.12	117	0.14
Poor	594	5.96	663	6.66	2158	7.63	2352	8.32	2273	5.05	2620	5.82	5025	6.04	5628	6.76
Good	5190	52.09	5275	52.94	13,293	47.00	14,152	50.04	20,535	45.63	20,985	46.63	39,018	46.87	40,487	48.63
Very good	4167	41.82	4013	40.27	12,795	45.24	11,741	41.52	22,138	49.19	21,329	47.40	39,100	46.97	37,015	44.46
Social characteristics																
Social support																
Low	432	4.34	447	4.49	2302	8.14	2399	8.48	3474	7.72	3418	7.59	6208	7.46	6277	7.54
Medium	5061	50.80	5219	52.38	13,000	45.97	13,183	46.62	19,428	43.17	19,193	42.65	37,489	45.03	37,669	45.25
High	4470	44.87	4297	43.13	12,978	45.89	12,698	44.90	22,101	49.11	22,392	49.76	39,549	47.51	39,300	47.21
Region																
Eastern	5096	51.15	5019	50.38	9601	33.95	8540	30.20	17,329	38.51	17,442	38.76	32,026	38.47	31,329	37.63
Central	2561	25.71	2531	25.40	6855	24.24	6657	23.54	10,362	23.03	10,165	22.59	19,778	23.76	19,211	23.08
Western	2306	23.15	2413	24.22	11,824	41.81	13,083	46.26	17,312	38.47	17,395	38.65	31,442	37.77	32,707	39.29
Residency																
Rural	2711	27.21	5789	58.10	7804	27.60	16,436	58.12	13,809	30.68	26,155	58.12	24,324	29.22	48,299	58.02
Urban	7252	72.79	4174	41.90	20,476	72.40	11,844	41.88	31,194	69.32	18,847	41.88	58,922	70.78	34,947	41.98
**Total**	**9963**	**100.00**	**9963**	**100.00**	**28,280**	**100.00**	**28,280**	**100.00**	**45,003**	**100.00**	**45,003**	**100.00**	**83,246**	**100.00**	**83,246**	**100.00**

Overall, 13% of respondents were older than 60 years, and more than half were female (55.61%), received tertiary education (56.39%), and were married (50.15%) at the time of the survey. About 36% were employed. Nearly half of the respondents (47.67%) fell into the lowest or lower quintile of per capita household income within their province or autonomous region. The vast majority resided in urban areas (70.78%), enrolled in the basic social health insurance (90.40%), and reported no chronic conditions (75.07%). Low levels of social support were rated by less than 10% of respondents.

Around 60% of respondents had a normal BMI in all three waves of surveys. The vast majority reported good or very good family health (> 90%) and were not smoking (85.78%) nor drinking alcohol (74.39%) at the time of the survey. The respondents reported higher levels of agreeableness (6.89 ± 1.45) and conscientiousness (6.68 ± 1.59) in personality features than others.

### Utility index

The weighted average UI score of respondents was highest in 2021, reaching 0.9363 (SD = 0.1389), before declining to 0.9332 (SD = 0.1524) in 2022 and further to 0.9244 (SD = 0.1546) in 2023, indicating a downward trend in HRQoL over time (β=-0.0143~-0.0081, *P* < 0.001). The most frequently reported problem was anxiety/depression (24.86%~28.03%), followed by pain/discomfort (24.02%~27.41%), mobility (11.06%~13.53%), usual activities (8.67%~11.23%), and self-care (6.59%~8.80%) (Supplemental file S3).

Pro-rich inequality in UI was evident as indicated by the positive value of EI, with lower UI scores being concentrated among those with lower income. The EI of UI decreased from 0.0703 in 2021 to 0.0635 in 2022 (β=-0.0105, *P* < 0.01), before bouncing back in 2023. However, the UI-EI composite index was the lowest in 2023 (92.4849), compared with that in 2021 (93.2993) and in 2022 (93.4837) (Table 2).

**Table 2:** Changes of EQ-5D-5 L utility index (UI) and Erreyger index (EI) over years (weighted) measured by regression coefficient (β)

Indicators		2021 (Ref)			2022			2023		
		Mean	SD	β	Mean	SD	β	Mean	SD	β
UI	Crude	0.9363	0.1389	-	0.9332	0.1524	-0.0081***	0.9244	0.1546	-0.0143***
	Adjusted for age, gender, residence.			-			-0.0036			-0.0130***
EI	Crude	0.0703	-	-	0.0635	-	-0.0105**	0.0747	-	-0.0065
	Adjusted for age, gender, residence.			-			-0.0104**			-0.0052
UI-EI index		93.2993	-	-	93.4837	-	-	92.4849	-	-

*: *p* < 0.05, **: *p* < 0.01, ***: *p* < 0.001

$$U - \,EI\,composite\, = \,\sqrt {(1 -|EI|*\,100\,*\,U\,*\,100)} $$

Overall, the respondents who had better health, higher socioeconomic status, higher health literacy, and supportive living environments tended to have higher UI but lower EI scores (Table 3).

**Table 3:** EQ-5D-5 L utility index (UI) (weighted mean ± standard deviation), Erreyger index (EI) scores and UI-EI index by characteristics of respondents

Variables	2021				2022				2023			
	UI	SD	EI	UI-EI	UI	SD	EI	UI-EI	UI	SD	EI	UI-EI
Individual physiological characteristics												
Gender												
Male	0.9414	0.1350	0.0660	93.7654	0.9308	0.1641	0.0643	93.3228	0.9249	0.1597	0.0734	92.5759
Female	0.9311	0.1426	0.0738	92.8610	0.9356	0.1393	0.0629	93.6337	0.9239	0.1492	0.0764	92.3754
Age (years)												
19–30	0.9594	0.1063	0.0351	96.2175	0.9256	0.1735	0.0632	93.1190	0.9406	0.1390	0.0443	94.8118
31–40	0.9663	0.0962	0.0315	96.7426	0.9410	0.1536	0.0529	94.4054	0.9446	0.1363	0.0503	94.7177
41–50	0.9551	0.1107	0.0427	95.6206	0.9462	0.1356	0.0522	94.6971	0.9473	0.1228	0.0536	94.6848
51–60	0.9397	0.1251	0.0631	93.8289	0.9526	0.1176	0.0543	94.9156	0.9288	0.1562	0.0466	94.1051
61–70	0.8911	0.1826	0.0976	89.6722	0.9326	0.1379	0.0562	93.8211	0.8969	0.1673	0.1048	89.6063
≥ 71	0.8465	0.2080	0.1574	84.4544	0.8674	0.1942	0.1109	87.8190	0.8334	0.2083	0.1507	84.1303
**Individual socio-economic characteristics**												
Educational attainment												
Illiteracy or elementary	0.8812	0.1926	0.1489	86.6045	0.9188	0.1525	0.0942	91.2256	0.8884	0.1832	0.1214	88.3523
Junior middle school	0.9424	0.1158	0.0541	94.4164	0.9485	0.1299	0.0546	94.6953	0.9347	0.1431	0.0567	93.8952
High school	0.9452	0.1240	0.0466	94.9305	0.9401	0.1475	0.0643	93.7885	0.9300	0.1496	0.0629	93.3525
Tertiary education	0.9571	0.1127	0.0320	96.2569	0.9306	0.1631	0.0562	93.7220	0.9364	0.1417	0.0552	94.0594
Marital status												
Unmarried	0.9462	0.1396	0.0511	94.7559	0.9168	0.1843	0.0718	92.2480	0.9264	0.1605	0.0651	93.0670
Married	0.9408	0.1289	0.0631	93.8856	0.9445	0.1297	0.0532	94.5649	0.9330	0.1399	0.0654	93.3796
Divorced/widowed	0.8554	0.2022	0.1664	84.4413	0.8586	0.2245	0.1366	86.0987	0.8325	0.2247	0.1584	83.7032
Employment status												
Employed	0.9666	0.0833	0.0251	97.0754	0.9544	0.1221	0.0347	95.9809	0.9445	0.1315	0.0481	94.8224
Student	0.9459	0.1400	0.0374	95.4194	0.9119	0.1925	0.0747	91.8603	0.9339	0.1520	0.0498	94.2016
Retired	0.9021	0.1698	0.0793	91.1338	0.9033	0.1692	0.0761	91.3542	0.8772	0.1859	0.1127	88.2204
Others	0.9139	0.1628	0.1148	89.9461	0.9327	0.1493	0.0859	92.3353	0.9221	0.1564	0.0905	91.5776
Basic social health insurance												
No	0.9214	0.1644	0.1018	90.9698	0.8898	0.2349	0.1164	88.6683	0.8968	0.2129	0.0652	91.5604
Yes	0.9400	0.1315	0.0622	93.8919	0.9369	0.1426	0.0591	93.8900	0.9262	0.1498	0.0752	92.5506
Commercial medical insurance												
No	0.9364	0.1387	0.0708	93.2793	0.9346	0.1504	0.0656	93.4499	0.9260	0.1507	0.0749	92.5532
Yes	0.9276	0.1531	0.0487	93.9357	0.9008	0.1896	0.0685	91.6044	0.8901	0.2204	0.1233	88.3375
Per capita monthly household income (Yuan)												
Lowest	0.9185	0.1598	-	-	0.9173	0.1740	-	-	0.9062	0.1680	-	-
Lower	0.9396	0.1307	-	-	0.9373	0.1326	-	-	0.9281	0.1455	-	-
Middle	0.9487	0.1297	-	-	0.9450	0.1337	-	-	0.9384	0.1367	-	-
Higher	0.9541	0.1077	-	-	0.9469	0.1349	-	-	0.9297	0.1542	-	-
Highest	0.9565	0.1032	-	-	0.9345	0.1643	-	-	0.9374	0.1550	-	-
**Individual health literacy**												
Health literacy												
Low	0.8126	0.2683	0.2006	80.5968	0.8431	0.2447	0.1395	85.1743	0.8949	0.1897	0.0986	89.8183
Medium	0.9312	0.1386	0.0695	93.0888	0.9303	0.1503	0.0647	93.2791	0.9405	0.1263	0.0578	94.1359
High	0.9678	0.0878	0.0303	96.8775	0.9589	0.1178	0.0303	96.4288	0.9319	0.1510	0.0623	93.4783
**Individual health behavior**												
Smoking												
No	0.9366	0.1378	0.0678	93.4420	0.9386	0.1452	0.0625	93.8087	0.9324	0.1414	0.0679	93.2293
Yes	0.9345	0.1443	0.0820	92.6201	0.9085	0.1792	0.0648	92.1741	0.8909	0.1970	0.1017	89.4595
Alcohol drinking												
No	0.9352	0.1398	0.0718	93.1701	0.9393	0.1400	0.0609	93.9221	0.9261	0.1520	0.0745	92.5818
Yes	0.9378	0.1376	0.0679	93.4948	0.9117	0.1876	0.0747	91.8492	0.9191	0.1622	0.0760	92.1550
**Individual health status**												
Chronic conditions												
0	0.9617	0.1037	0.0365	96.2619	0.9513	0.1352	0.0436	95.3848	0.9590	0.1133	0.0391	95.9939
1	0.8976	0.1705	0.1003	89.8681	0.9144	0.1577	0.0697	92.2304	0.9042	0.1622	0.0696	91.7235
2 or more	0.8217	0.2071	0.1699	82.5878	0.8465	0.2062	0.1307	85.7862	0.8244	0.2149	0.1514	83.6385
Body mass index (BMI)												
Underweight	0.9157	0.1652	0.0662	92.4681	0.9143	0.1832	0.0831	91.5630	0.9031	0.1889	0.1046	89.9242
Normal	0.9424	0.1293	0.0663	93.8057	0.9354	0.1476	0.0601	93.7652	0.9290	0.1450	0.0667	93.1139
Overweight	0.9307	0.1470	0.0776	92.6509	0.9387	0.1455	0.0640	93.7363	0.9260	0.1528	0.0777	92.4116
Obesity	0.9311	0.1465	0.0860	92.2506	0.9337	0.1409	0.0502	94.1692	0.9055	0.1882	0.0888	90.8332
**Family characteristics**												
Family health												
Very poor	0.6564	0.3455	0.4655	59.2350	0.8818	0.1820	0.1680	85.6541	0.7783	0.3077	0.3647	70.3134
Poor	0.8815	0.2131	0.1136	88.3938	0.8442	0.2314	0.1068	86.8356	0.8001	0.2835	0.1287	83.4938
Good	0.9338	0.1421	0.0714	93.1212	0.9248	0.1687	0.0711	92.6845	0.9129	0.1661	0.0818	91.5530
Very good	0.9494	0.1122	0.0564	94.6495	0.9613	0.0912	0.0393	96.0976	0.9515	0.1013	0.0499	95.0800
**Social characteristics**												
Social support												
Low	0.8939	0.1955	0.1000	89.6973	0.8616	0.2281	0.1061	87.7611	0.8538	0.2512	0.1328	86.0476
Medium	0.9266	0.1517	0.0761	92.5254	0.9208	0.1605	0.0710	92.4885	0.9156	0.1563	0.0832	91.6231
High	0.9524	0.1107	0.0564	94.7951	0.9596	0.1154	0.0428	95.8372	0.9427	0.1277	0.0553	94.3688
Region												
Eastern	0.9387	0.1387	0.0610	93.8857	0.9234	0.1608	0.0516	93.5856	0.9189	0.1643	0.0658	92.6521
Central	0.9327	0.1453	0.0854	92.3573	0.9350	0.1530	0.0623	93.6307	0.9369	0.1331	0.0760	93.0418
Western	0.9351	0.1323	0.0754	92.9808	0.9386	0.1460	0.0776	93.0477	0.9226	0.1558	0.0876	91.7487
Residency												
Rural	0.9287	0.1502	0.0888	91.9888	0.9275	0.1626	0.0800	92.3732	0.9168	0.1676	0.0844	91.6208
Urban	0.9468	0.1207	0.0460	95.0408	0.9411	0.1365	0.0430	94.9011	0.9349	0.1337	0.0593	93.7780
**Total**	**0.9363**	**0.1389**	**0.0703**	**93.2993**	**0.9332**	**0.1524**	**0.0635**	**93.4837**	**0.9244**	**0.1546**	**0.0747**	**92.4849**

**Fig. 2:**
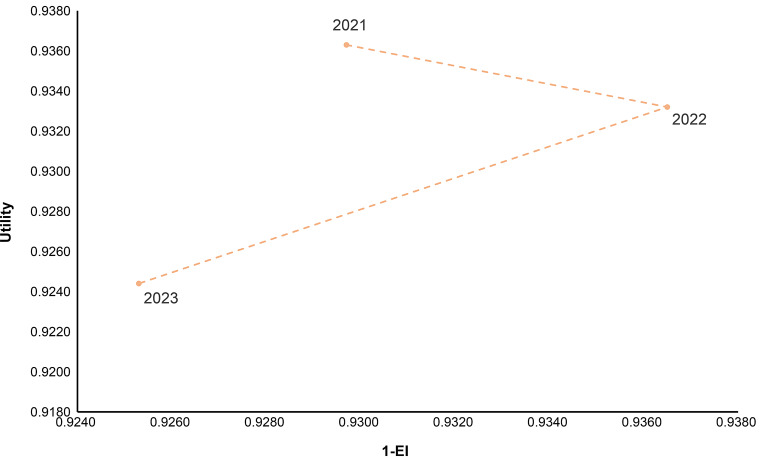
The EQ-5D-5 L utility index and Erreyger Index (EI) by year

Male respondents had higher UI, lower EI scores and UI-EI composite index than their female counterparts in 2021 and 2023, but this was reversed in 2022. The UI scores declined by age, but the EI of UI scores increased by age. And the UI-EI composite index has the similar trend with the UI scores (Fig. 2).

The eastern developed and western under-developed zones had an upward trend in UI scores over the three years, compared with a downward trend in the central developing zone. The lowest EI was also found in the eastern developed zone. Meanwhile, the western under-developed zone experienced an upward trend in EI. The UI-EI composite index in the eastern region showed a decreasing trend, while the central and western regions showed a fluctuating trend during this three-years period. And in 2021, the UI-EI composite index was highest in the eastern region, while in 2022 and 2023, the central region had the highest UI-EI composite index.

Rural respondents had lower UI but higher EI scores than their urban counterparts. During these three years, the UI-EI composite index in urban areas consistently remained higher than in rural areas.

### Determinants of utility index

The OLS regression showed that UI decreased by age (β=-0.0553~-0.0093, *P* < 0.05). Those who were married (β = 0.0180, *P* < 0.001), had a highest qualification of junior middle school (β = 0.0072, *P* < 0.05), were covered by basic health social insurance (β = 0.0155, *P* < 0.001), resided in urban (β = 0.0049, *P* < 0.001) and the western underdeveloped zone (β = 0.0044, *P* < 0.01), and reported higher health literacy (β = 0.0228 ~ 0.0257, *P* < 0.001) and higher social support (β = 0.0222 ~ 0.0300, *P* < 0.001) had higher UI scores than others. By contrast, being female (β=-0.0071, *P* < 0.001), smoking (β=-0.0196, *P* < 0.001), drinking alcohol (β=-0.0107, *P* < 0.001), and living with chronic conditions (β=-0.0989~-0.0417, *P* < 0.001) were associated with lower UI scores. Higher income was associated with higher UI scores (β = 0.0077 ~ 0.0123, *P* < 0.01), indicating pro-rich inequality (Table 4).

**Table 4:** Decomposition of contributors to EQ-5D-5 L utility index (UI) and Erreyger index (EI) scores (weighted)

Variables	UI				EI	
	OLS		Shapley decomposition		RIF-EI-OLS	
	Coef.	*P* value	Shapley value	con %	Coef.	*P* value
**Individual physiological characteristics**			**0.0117**	**7.15%**		
Gender (Ref: male)						
Female	-0.0071	0.0000			-0.0085	0.0030
Age (years) (Ref: 19–30)						
31–40	-0.0114	0.0010			0.0093	0.1330
41–50	-0.0102	0.0080			0.0019	0.7850
51–60	-0.0093	0.0160			-0.0048	0.4910
61–70	-0.0146	0.0010			0.0091	0.2570
≥ 71	-0.0553	0.0000			0.0471	0.0000
**Individual socio-economic characteristics**			**0.0183**	**11.15%**		
Educational attainment (Ref: illiteracy or elementary)						
Junior middle school	0.0072	0.0030			-0.0229	0.0000
High school	0.0005	0.8640			-0.0138	0.0020
Tertiary education	-0.0073	0.0070			-0.0093	0.0640
Marital status (Ref: unmarried)						
Married	0.0180	0.0000			-0.0043	0.5030
Divorced/widowed	-0.0284	0.0000			0.0220	0.0330
Employment status (Ref: employed)						
Student	-0.0180	0.0000			0.0068	0.0530
Retired	-0.0088	0.0040			-0.0120	0.0840
Others	-0.0046	0.0170			-0.0191	0.0000
Basic social health insurance (Ref: no)						
Yes	0.0155	0.0000			-0.0002	0.9700
Commercial medical insurance (Ref: no)						
Yes	-0.0204	0.0000			-0.0222	0.0170
Household income ranking (Ref: lowest)						
Lower	0.0101	0.0000			-0.0189	0.0000
Middle	0.0123	0.0000			-0.0130	0.0000
Higher	0.0090	0.0000			-0.0223	0.0000
Highest	0.0077	0.0020			-0.0189	0.0030
**Individual health literacy**			**0.0077**	**4.68%**		
Health literacy (Ref: low)						
Medium	0.0228	0.0000			-0.0116	0.0050
High	0.0257	0.0000			-0.0105	0.0400
**Individual health behavior**			**0.0069**	**4.20%**		
Smoking (Ref: no)						
Yes	-0.0196	0.0000			-0.0050	0.2440
Alcohol drinking (Ref: no)					0.0027	0.4370
Yes	-0.0107	0.0000				
**Individual health status**			**0.0604**	**36.82%**		
Chronic conditions (Ref:0)						
1	-0.0417	0.0000			-0.0031	0.3470
2 or more	-0.0989	0.0000			0.0315	0.0000
Body mass index (BMI) (Ref: underweight)						
Normal	0.0120	0.0000			-0.0080	0.0850
Overweight	0.0105	0.0000			-0.0078	0.1340
Obesity	0.0035	0.4080			-0.0184	0.0160
Extraversion	0.0018	0.0000			-0.0001	0.8540
Agreeableness	0.0020	0.0000			-0.0005	0.5590
Conscientiousness	0.0012	0.0140			0.0023	0.0060
Nervousness	-0.0055	0.0000			0.0020	0.0200
Openness	-0.0001	0.8700			0.0006	0.4050
**Family characteristics**			**0.0259**	**15.81%**		
Family health (Ref: very poor)						
Poor	0.0330	0.4010			-0.1349	0.0000
Good	0.0916	0.0190			-0.1319	0.0000
Very good	0.1096	0.0050			-0.1297	0.0010
**Social characteristics**			**0.0099**	**6.03%**		
Social support (Ref: low)						
Medium	0.0222	0.0000			-0.0023	0.7580
High	0.0300	0.0000			-0.0018	0.8120
Region (Ref: eastern)						
Central	0.0028	0.1030			0.0134	0.0000
Western	0.0044	0.0060			0.0112	0.0000
Residency (Ref: rural)						
Urban	0.0049	0.0000			0.0031	0.2520
**Year (Ref: 2021)**			**0.0009**	**0.55%**		
2022	-0.0045	0.0480			-0.0040	0.3140
2023	-0.0022	0.2960			-0.0068	0.0710

The Shapley decomposition analysis showed that individual health was the largest contributor, accounting for 36.82% of variations in UI scores. This was followed by family characteristics (15.81%) and individual socio-economic characteristics (11.15%). The other determinants accounted for less than 10% of variations in UI scores for each category. The variable of year contributed to only 0.55% of variations in UI scores (Table 4).

The RIF-EI-OLS model showed that inequality in UI scores was explained by older age (β = 0.0471 for those older than 70 years, *P* < 0.001), marriage disruption (β = 0.0220 for those divorced or widowed, *P* < 0.05), and living with two or more chronic conditions (β = 0.0315, *P* < 0.001). By contrast, higher educational attainment (β=-0.0093~-0.0229, *P* < 0.1), better health literacy (β=-0.0116~-0.0105, *P* < 0.05) and having a good family health (β=-0.1349~-0.1297, *P* < 0.001) contributed negatively to inequality in UI scores. Conscientiousness (β = 0.0023, *P* < 0.05) and nervousness (β = 0.0020, *P* < 0.05) personality was associated with higher health inequality. Overall, increased income was associated with decreased inequality in UI scores (β=-0.0223~-0.0130, *P* < 0.01). Inequality in UI scores also varied across regions, with the central developing (β = 0.0134, *P* < 0.001) and the western underdeveloped zones (β = 0.0112, *P* < 0.001) featuring higher inequality compared with their eastern developed counterpart (Table 4).

### EQ-VAS scores

Similar results for the EQ-VAS scores were found. The EQ-VAS scores showed a significant decreasing trend over time (β=-8.3268~-7.8634, *P* < 0.001). However, the EI of EQ-VAS exhibited an upward trend (β = 0.0172 ~ 0.0191, *P* < 0.05), indicating increasing inequality (Supplementary materials S4). Higher socioeconomic status, better health, higher health literacy, and supportive living environments were associated with higher EQ-VAS and lower EI scores (Supplementary materials S5). Senior age (> 70 years) and living with chronic conditions were associated with higher inequality in EQ-VAS, while higher health literacy, better family health, and higher social support were associated with lower inequality in EQ-VAS (Supplementary materials S6).

## Discussion

This study examined trends in HRQoL from both a level and equity perspective, utilizing nationwide data from mainland China between 2021 and 2023. Our findings reveal a slight declining trend in both EQ-UI and EQ-VAS scores from 2021 to 2023, consistent with patterns observed in previous global studies [18, 51, 52]. However, the changes in UI scores (effect size = -0.058 to -0.022, both < 0.5) may not be of significant clinical importance [17, 53]. The mean weighted UI scores for the Chinese population ranged from 0.9244 to 0.9363 during this period, closely aligning with the 0.939 score reported in Tianjin in 2020 [54].

We observed an increase in the percentage of reported problems across the five dimensions of HRQoL, with pain/discomfort and usual activities showing the greatest change. Among these, anxiety/depression emerged as the most commonly reported issue, followed by pain/discomfort, which is consistent with findings from a study in China [55]. During the survey period, China implemented a strict “zero-COVID” or “dynamic zero-COVID” strategy, which included large-scale nucleic acid testing and lockdown measures in areas with outbreaks [20]. These prolonged measures restricted daily life and social interactions, potentially contributing to emotional distress and a decline in HRQoL [56].

The pro-rich inequality in HRQoL persisted in mainland China according to our study, with the EI for UI ranging from 0.0635 to 0.0747, which is consistent with previous studies conducted in China [3, 9, 32]. The EI in mainland China is higher than in Thailand [57] (0.024 in 2019) and Chile [58] (0.047 in 2013), but is much lower than Argentina [59] (0.1223 in 2013) and Iran [60] (0.133 in 2016). Despite the Chinese government’s efforts over the past few decades to narrow the wealth gap and rural-urban health disparity, concerns remain [61].

Despite the decline in EQ-UI scores between 2021 and 2023, inequality in UI decreased, as reflected by the EI values in 2022 and 2023—findings that are consistent with those of previous studies [19, 48]. However, the EI of EQ-VAS suggests a rise in health inequality over the same period. This discrepancy may indicate that the EQ-UI does not fully capture certain concerns reflected in residents’ VAS ratings, in particular among those with relatively lower income who were most impacted by the pandemic. Nonetheless, it remains essential to monitor the long-term trajectory of health inequality and implement targeted policy interventions to address disparities related to region, urban–rural residence, and income in China.

Overall, 2023 recorded the lowest composite index score, considering both UI and EI, compared to 2021 and 2022. Numerous factors contributed to changes in HRQoL and health inequalities, with income, health literacy, family health, chronic conditions, and residency identified as key determinants in our study. Globally, the Universal Health Coverage (UHC) service coverage index (SCI) has been increasing since 2000; however, the COVID-19 pandemic poses a significant threat to reversing two decades of progress [2].

Our research indicated that higher income can be a crucial factor in improving HRQoL and mitigating health inequalities, as supported by multiple studies [6, 29, 32]. Income also shapes health-seeking behaviors, health expectations, and other determinants of health [62, 63]. Unfortunately, evidence has shown that the COVID-19 pandemic has led to widespread income losses globally [64], further jeopardizing the health outcomes associated with COVID-19 [65, 66].

Education has been shown to improve HRQoL and promote health equity, as supported by previous studies [67, 68]. Individuals with higher educational attainment generally have better health literacy, greater access to health information [69], and more stable, higher-paying jobs, enabling families to accumulate health resources [70] that can buffer against health risks during the COVID-19 pandemic. Furthermore, the disruption of formal education during the pandemic disproportionately affected students from lower socioeconomic backgrounds, potentially exacerbating existing health inequalities [71].

We found that higher health literacy is a significant factor in increasing HRQoL and reducing health inequalities, consistent with previous studies [32, 72]. Higher health literacy is associated with healthier behaviors, including greater individual engagement in healthcare [32, 73–75]. Conversely, lower health literacy is often linked with lower income [76]. The COVID-19 pandemic has underscored the importance of health literacy [74], particularly in combating misinformation and disinformation [77].

Regional and urban-rural health disparities have long been a concern in China, and this study further verifies their continuous existence. Urban residents have higher HRQoL but lower health inequality compared with their rural counterparts. The higher health inequality in rural China is likely associated with a lack of resources and weaker healthcare systems in these areas [48]. The eastern zone of mainland China, characterized by higher socioeconomic development, shows lower HRQoL but also lower health inequality, consistent with previous studies [9, 32, 48]. In contrast, the less developed zones exhibit higher health inequalities. These regional disparities are likely due to the greater levels of welfare and infrastructure support available in the eastern zone, although chronic conditions have become a more significant health concern there [78]. During the COVID-19 pandemic, regional disparities in health appear to have been reduced thanks to enhanced resource support from the government.

The findings of this study carry important policy implications and offer valuable insights. However, it is important to recognize that some of these findings may not be directly applicable to other countries due to China’s unique national circumstances. Despite rapid economic development and significant strengthening of its health system, income-related health inequality remains a major concern, and regional and urban-rural disparities persist, though some improvements have been made. The government should allocate more resources to disadvantaged populations, particularly those with lower income and educational attainment and those burdened by chronic conditions. Previous studies have shown that catastrophic health expenditure remains a significant concern for these groups in China [79].

### Limitations

There are some potential limitations in this study worth noting. First, the 2021 PBICR does not allow for a precise breakdown of age, as individuals aged 12 to 18 years are grouped together. Therefore, the conclusions of this study may not be generalizable to all age populations. Second, the data do not capture potential behavioral changes related to COVID-19 policies, such as lockdown measures and region-specific policy adaptations. Additionally, the study may not account for all underlying factors affecting HRQoL and health inequality. Finally, the sampling methods resulted in an overrepresentation of the western underdeveloped zone, although we adjusted the results using population weights. As this study was conducted during the COVID-19 pandemic, future research should investigate the long-term trends in health inequality.

It is important to recognize that different health measurement instruments may yield varying results. Overall, the EQ-VAS results (Supplemental Files S3–S5) align with the patterns observed in the UI results. However, discrepancies emerge in the temporal trends of the EI when comparing findings based on UI and VAS. A potential explanation lies in the conceptual differences between the two measures: the VAS reflects individuals’ subjective evaluations of their general health status, which may be influenced by personal expectations and perceptions, whereas the UI captures health status based on standardized population-level valuations. As a result, individual assessments may not always align with values endorsed at the population level.

## Conclusion

During the COVID-19 pandemic, residents of mainland China experienced a slight decline in HRQoL, alongside a reduction in UI inequality but an increase in VAS inequality in 2022 and 2023. Overall, 2023 recorded the lowest composite index score, which accounts for both UI and EI, compared to 2021 and 2022. Several key factors contributed to these changes, including socioeconomic status, chronic conditions, health literacy, and family health. Despite significant efforts by the Chinese government to develop infrastructure and expand universal health coverage, the pandemic may have hindered progress toward achieving healthcare equality.

## Electronic supplementary material

Below is the link to the electronic supplementary material.

Supplementary Material 1

## Data Availability

Data are available on reasonable request. Data are not publicly available but may be requested from the authors on reasonable request. Data used in this study were extracted from the Psychology and Behavior Investigation of Chinese Residents (PBICR) conducted by the corresponding author Y W.
